# Rural Kansas Family Physician Satisfaction with Caring for Spanish-Speaking Only Patients

**Published:** 2017-11-30

**Authors:** Theresia Neill, Gretchen Irwin, C. Scott Owings, William Cathcart-Rake

**Affiliations:** 1 University of Kansas School of Medicine-Salina; 2 University of Kansas School of Medicine-Wichita, Department of Family and Community Medicine, Family Medicine Residency Program at Wesley Medical Center

**Keywords:** language barrier, Hispanics, job satisfaction, rural health services

## Abstract

**BACKGROUND:**

Patient satisfaction with the care they receive can be influenced negatively by a language barrier between the physician and patient. However, there is a paucity of information regarding the consequences of a language barrier on physician satisfaction, although this barrier has the potential to decrease physician wellness. This study sought to determine if a language barrier is a source of professional dissatisfaction in family medicine physicians in rural Kansas.

**METHODS:**

In a cross-sectional study, members of the Kansas Academy of Family Physicians who practiced in the rural Kansas counties with the highest percentage of Hispanic residents were surveyed. A questionnaire was developed to determine the demographics of the physician, details regarding his or her practice, and percentage of Hispanic and Spanish-speaking only (SSO) patients in their practice. Physicians also were queried as to their level of Spanish-speaking ability, availability of certified interpreters, and their satisfaction with caring for their SSO patients.

**RESULTS:**

Fifty-two physicians were identified and sent questionnaires by mail. Eighteen questionnaires were completed and returned, resulting in a 34% response rate. Respondents remained anonymous. In the practices surveyed, 61% of practice settings had a Hispanic-patient population greater than 25%. Only one of the eighteen respondents had greater than 25% of SSO patients in his or her practice. A certified interpreter was used less than 25% of the time in over 75% of the clinical encounters with SSO patients. Seventy-five percent of physicians reported no difficulty establishing trust and rapport with their SSO patients. Eighty-nine percent of respondents rated their relationship with SSO patients as good to excellent, and 83% were satisfied with the care they were able to provide this group. Seventy-eight percent of respondents reported that their ability to care for SSO patients decreased or had no effect on their professional satisfaction. Seventy-eight percent of physicians also rated their overall professional satisfaction in regards to their physician/patient relationship as good to excellent. However, language barriers affected physician-patient relationships, physician satisfaction with care, and professional satisfaction.

**CONCLUSION:**

Language barrier affected physician’s relationships with SSO patients, led to decreased physician satisfaction with the care they provided and to decreased professional satisfaction.

## Introduction

With so much emphasis on improving physician wellness and satisfaction, it is important to determine the factors that affect these elements. RAND Health identified that an important factor influencing physician satisfaction is the physician’s perception about quality of care they deliver.^1^ A physician’s ability to deliver high-quality patient care was an important source of his or her professional satisfaction. There are many factors interfering with a physician’s ability to deliver quality care, causing frustration for the physician and leading to a decrease in physician professional satisfaction. If this frustration stems from poor communication with patients, impediments to care can be significant and outcomes can suffer. Physicians and patients both suffer when language barriers exist.

Kansas has seen an increase in the Hispanic population, especially in rural communities, over recent decades, with a 59.4% increase overall from 2000 to 2010.^2^ The significant increase in the Hispanic population has led to many SSO patients seeking medical providers in these rural communities. Often, these communities do not have an on-site interpreter and may rely on a communication device or other resources, such as bilingual staff or family of the patient.

Cultural, personal beliefs, values, and language differences influence patient satisfaction.^3^–^5^ Hispanics are more likely to report dissatisfaction with their physician relationship, have less continuity of care, and perceive poorer quality of care.^3^ Patients who use an interpreter or do not have an interpreter when one is necessary are not as satisfied with the patient-provider relationship.^6^ Clinicians reported that communication difficulties affect their ability to treat and connect with their patients and evidence showed that race, ethnicity, and language have a substantial influence on the quality of the physician-patient relationship.^7^,^8^ Language barriers between physicians and patients also can reduce patient compliance and quality of care.^9^

The aim of this study was to determine if a language barrier was a source of professional dissatisfaction in family medicine physicians in rural Kansas. The authors hypothesized that rural Kansas physicians would be less satisfied caring for SSO patients. Recognition of this factor, which could affect physician wellness negatively, could be a first step in seeking resolution to the problem.

## Methods

In a cross-sectional study, fifty-two members of the Kansas Academy of Family Physicians practicing in the fifteen rural Kansas counties with the highest percentage of Hispanic residents were invited to complete a seventeen question survey. Counties with the highest percentage of Hispanic patients were Seward (59%), Ford (53%), Finney (48%), Grant (46%), Stanton (36%), Stevens (35%), Hamilton (34%), Kearney (30%), Haskell (29%), Wichita (28%), Lyon (21%), Morton (21%), Edwards (20%), Scott (18%), and Greeley (18%).^10^

A questionnaire (Appendix A) was developed to determine the demographics of the physician and details about his or her practice, including years in practice, practice setting, and percentage of Hispanic and SSO patients in their practice. Questions also were asked about the physician’s level of Spanish-speaking ability, availability of certified interpreters, and the physician-patient relationship with SSO patients. Physicians were asked to rate their ability to provide care to their patients and the satisfaction with the care that they delivered. They also were asked to distinguish this from their overall professional satisfaction in regards to their relationship with SSO patients. Respondents remained anonymous.

Descriptive analysis methods were used to determine details about the survey respondents. Pearson’s correlation analysis was used to assess associations between different survey response items.^11^ All statistical analyses were performed using SPSS for Windows, version 23.

## Results

Eighteen questionnaires were returned from the 52 physicians sent questionnaires, resulting in a 34% response rate. Respondent demographics are shown in Table 1. Seventeen (94%) of the physician respondents self-identified as white and two (11%) respondents had a Hispanic, Spanish, or Latino heritage. Practice descriptions are noted in Table 2. Of the eighteen physician respondents, seven engaged in private practice (solo, small group, medium to large group), while the remainder were hospital employees or worked for a Federally Qualified Health Center (FQHC), rural health clinic, or other safety-net clinic. One respondent was retired, and his or her responses were included in the analysis.

Eleven physician practices had a Hispanic-patient population greater than 25% (Table 3). SSO patients comprised greater than 25% of the patients in only one practice (Table 4). Private practices had significantly fewer patients who identified as Hispanic or Latino [correlation analysis: r(17) = .49, p = .05] and significantly fewer SSO patients [r(18) = .62, p < .01]. A decrease in the number of patients who identified as Hispanic or Latino in the physician’s clinic correlated with a more negative relationship with SSO patients [r(17) = −.53, p = .02] and a decrease in professional satisfaction caring for this population [r(17) = −.56, p < .01].

If physicians were Hispanic or Latino, their perception of their ability to care for SSO patients was not an issue [r(18) = −.52, p =.03]. Ten (56%) physicians claimed to have basic Spanish-speaking ability, six (33%) noted good to advanced Spanish-speaking ability, and two (11%) had no fluency in Spanish (Table 1). If physicians did not speak Spanish, their perception of their professional satisfaction with SSO patients was negative [r(18) = −.49, p = .04], and, if they spoke Spanish, their perception of their ability to care for SSO patients was positive [r(18) = .59, p = .01]. Certified interpreters were underutilized during clinic visits. A certified interpreter was used less than 25% of the time in over 75% of the clinical settings (Table 5).

Physician perceptions of his or her relationship with SSO patients and this effect on professional satisfaction are compiled in Table 6. Twelve physicians (67%) reported no difficulty establishing trust and rapport with their SSO patients, and sixteen (89%) rated their relationship with SSO patients as good to excellent. A better relationship (i.e., trust and rapport) with SSO patients correlated with increased professional satisfaction for the physician provider [r(18) = .91, p < .001]. Fifteen (83%) respondents were satisfied with the care they were able to provide to their SSO patients. Seventy-eight percent of respondents (14 physicians) also reported that their ability to care for SSO patients either decreased or had no effect on their professional satisfaction, while four physicians noted an increase in their professional satisfaction. Fourteen physicians (78%) also rated their overall professional satisfaction in regards to their physician/patient relationship as good to excellent. The level of satisfaction with the care provided to SSO patients correlated with a higher professional satisfaction for the provider [r(18) = .47, p<.05].

## Discussion

A major limitation of this study was the small number of physicians completing the survey. Repeated requests for responses and/or identifying a larger number of physicians in the fifteen Kansas counties studied may have increased the study’s power; however, in a study examining questionnaire response rates from individuals Baruch and Holtom^12^ noted that incentives or repeated reminders to participate in a survey did not significantly improve response rates. The authors also found the average response rate from individuals was 52.7% with a standard deviation of 20.4%. Although the number of responses in our study was less than hoped, the response rate was certainly within the predicted range for such a survey.

Although the questionnaire contained only five questions (#13–17) directly relating to physician relationships with SSO patients, the questions were deemed sufficient to gauge physician sentiments. A longer survey may have resulted in an even lower response rate. The vast majority of responding physicians established trust and rapport with SSO patients, rated their relationship with this patient cohort as good to excellent, and were satisfied with the care delivered to SSO patients. Despite its limitations, the data provided insight into rural physician satisfaction with caring for SSO patients, although the results may not be extrapolated outside of rural areas or to areas with greater resources available for SSO patients.

Private practices had significantly fewer SSO patients than other practices. Consequently, fewer SSO patients in a physician’s practice were correlated with a more negative physician-patient relationship, which led to a decreased professional satisfaction overall. Understandably, those physicians who do not interact with this population of patients as often as other physicians are less comfortable with the infrequent interactions or do not have the processes in place to address this patient population’s needs.^6^

If a physician had Hispanic or Latino background or spoke Spanish, his or her perception of the ability to care for SSO patients increased. Conversely, if a physician did not speak Spanish, his or her perception of professional satisfaction caring for SSO patients decreased. Use of a certified interpreter might improve physician-patient communication, but engaging the services of a certified interpreter was underutilized by the respondents in this study. Employing an interpreter could be an area of improvement in rural practices to improve the relationship with Spanish-speaking only patients and, in turn, increase physician satisfaction.

## Conclusions

One of the most important factors for physician satisfaction is the delivery of high quality care to patients.^1^,^13^ Language barriers can interfere with the quality of care a physician provides his or her patients. Language barriers not only impact physician-patient relationships, including the physician’s understanding of the patient’s symptoms and the patient’s understanding of the physician’s diagnoses and treatment recommendations, but can cause decreased physician satisfaction with the level of care provided and decreased professional satisfaction. Fortunately, the majority of the physician respondents in this study were satisfied with the care they delivered to SSO patients. However, this study also provided evidence that caring for SSO patients by physicians with limited encounters and/or no or minimal ability to converse in Spanish may be a significant source of physician dissatisfaction. Recognition of this issue and developing means to assist this group of physicians could improve patient care and physician well-being. Given the growing Hispanic population and SSO population in Kansas, it is imperative that ways to dismantle language barriers be explored and implemented. Possible ways to address the language barrier is through increased utilization of certified interpreters, tools to start the conversation with SSO patients (Appendix B), training of minority physicians, and training in medical Spanish for physicians.^8^,^9^

## Figures and Tables

**Table 1:** Demographics of the rural Kansas family medicine physicians surveyed.

	Number (%)
**Gender**	
Male	13 (72%)
Female	5 (28%)
**Age**	
30–39	8 (44%)
40–49	4 (22%)
50–59	0 (0%)
>60	6 (33%)
**Race**	
White/non-Hispanic	15 (83%)
White/Hispanic	2 (11%)
Asian	1 (6%)
**Spanish-speaking ability**	
None	2 (11%)
Basic ability	10 (56%)
Good to advanced ability	6 (33%)

**Table 2:** Practice description of rural physicians surveyed.

Solo Practice	1
Small Group Practice	5
Medium to Large Group Practice	1
Hospital Employed	7
FQHC, etc.	3
Other (retired)	1

**Table 3:** Hispanic patients in practice.

Number (%) of Practices	% Hispanic Patients
7 (39%)	10–25%
9 (50%)	26–50%
2 (11%)	51–75%

**Table 4:** Percent Spanish-speaking only Hispanic patients.

Number (%) of Practices	% Spanish-speaking Only Patients
5 (28%)	<10%
12 (67%)	10–25%
1 (5%)	26–50%

**Table 5:** Use of certified interpreter for clinical visits for Spanish-speaking only patients.

% of Encounters for All Practices Surveyed	% of Time Interpreter Was Used
78%	<25%
5%	26–50%
11%	51–75%
6%	>75%

**Table 6:** Physician perceptions regarding their relationship with SSO patients.

Difficulty Establishing Trust & Rapport	

Yes	4 (22%)
No	12 (67%)
Did Not Answer	2 (11%)

Rating of Relationship	

Poor	0 (0%)
Fair	2 (11%)
Good	10 (56%)
Excellent	6 (33%)

Physician Satisfied with Care Provided	

Yes	15 (83%)
No	3 (17%)

Ability to Care for SSO Patient Effected Professional Satisfaction	

No Effect	6 (33%)
Decreased Satisfaction	8 (44%)
Increased Satisfaction	4 (22%)

Professional Satisfaction in Regards to Physician/Patient Relationship	

Poor	0 (0%)
Fair	4 (22%)
Good	8 (44%)
Excellent	6 (33%)

## Appendix A Questionnaire: Study on Relationships between Physicians and Spanish-Speaking Patients

The purpose of this questionnaire is to assess physician satisfaction related to the care of Spanish-speaking patients in your practice. Your answers will be kept anonymous; you will only be identified by study personnel through your demographic information and results will be presented only in the aggregate. The questionnaire should take less than 5 minutes to complete. Please return in the provided envelope. Thank you for participating in this questionnaire!

1. How do you identify your gender?
  - Male
  - Female
  - Other- please explain:_______
2. What is your age?
  - <30 years
  - 30–39 years
  - 40–49 years
  - 50–59 years
  - >60 years
3. What is your race?
  - White
  - Black or African American
  - Asian/Pacific Islander
  - American Indian
  - Alaskan Native
  - Other- please explain:_______
4. Are you of Hispanic, Spanish or Latino origin?
  - Yes
  - No
5. How do you assess your level of Spanish speaking ability?
  - None
  - Basic: I speak the language imperfectly and only to a limited degree and in limited situations. I have difficulty in or understanding extended conversations. I am unable to understand or communicate most healthcare concepts.
  - Fair: I speak and understand well enough to have extended conversations about current events, work, family, or personal life. Native speakers notice many errors in my speech or understanding. I have difficulty communicating about healthcare concepts.
  - Good: I speak well enough to participate in most conversations. Native speakers notice some errors in my speech or understanding, but my errors rarely cause misunderstanding. I have some difficulty communicating necessary health concepts.
  - Advanced: I speak very accurately, and I understand other speakers very accurately. Native speakers have no problem understanding me, but they probably perceive that I am not a native speaker.
  - Native/functionally native: I converse easily and accurately in all types of situations. Native speakers, including the highly educated, may think that I am a native speaker, too.
6. Are you trained as a M.D. or D.O?
  - M.D.
  - D.O.
7. How many years have you been in practice outside of residency?
  - <5 years
  - 5–10 years
  - 10–20 years
  - >20 years
8. What is your current practice situation?
  - Solo private practice
  - Small group private practice
  - Medium to large group private practice
  - Hospital employed
  - FQHC, rural health clinic, other safety-net clinic
  - Other- please explain:_________
9. What percentage of your patients identify as Hispanic? If unsure, please estimate best guess.
  - <10%
  - 10–25%
  - 26–50%
  - 51–75%
  - >75%
10. What percentage of your Hispanic patients are Spanish-speaking only? If unsure, please estimate best guess.
  - <10%
  - 10–25%
  - 26–50%
  - 51–75%
  - >75%
11. What resources do you have for interpretive services?
  - Electronic device (i.e., iPad)
  - Telephone service
  - In person certified translator
  - Bilingual medical personnel not certified for interpretation
  - Family or friends of patient
  - Other- please explain:_________
12. How often is a certified interpreter used with exclusively Spanish-speaking only patients?
  - <25% of the time
  - 25–50% of the time
  - 51–75% of the time
  - 100% of the time
13. Do you find that having a language barrier with your Spanish speaking patients makes it more difficult to establish rapport and trust?
  - Yes
  - No
14. How would you rate your relationship with your Spanish-speaking only patients?
  - Excellent
  - Good
  - Fair
  - Poor
15. Are you satisfied with the care you are able to provide to your Spanish-speaking only patients?
  - Yes
  - No
16. How does your ability to care for your Spanish-speaking only patients affect your professional satisfaction?
  - No affect
  - Professional satisfaction decreased
  - Professional satisfaction increased
17. How would you rate your professional satisfaction in regards to your physician/patient relationship with Spanish-speaking only patients?
  - Excellent
  - Good
  - Fair
  - Poor

## Appendix B Start the Conversation

**Table:**

Comenzar la Conversación/Start the Conversation	
This tool is to be used by the physician to start the conversation with a Spanish speaking-only patient while waiting for the interpreter.	
**Phrase** (physician speaking to patient)	**Translation**
Hola! Me llamo Doctor(a) (insert last name). **Doctor = male, Doctora = female**	Hello! My name is Doctor (insert last name here).
Soy su doctor(a) hoy. Estoy esperando la intérprete.	I will be your doctor today. I am waiting for the interpreter.
Estoy usando una persona/el iPad/el teléfono para interpretación hoy. Será solo unos minutos.	I will be using a person/the iPad/the telephone for interpretation today. It will be just a few minutes.
